# Account of an Operation for the Removal of an Ovarian Tumor

**Published:** 1850-06

**Authors:** 


*Account of an operation for the removal of an Ovarian Tumor.* By ALDEN MARCH, M. D., President, and Professor of Surgery in the Albany Medical College.

On Friday the 7th of December, 1849, the wife of Mr. W. J. P., of Granby, Mass., aged 49 years, was brought to me in company with her husband and her family physician, Dr. Alexander Le Baron Munroe, of the same place, for consultation and advice in what was supposed to be an *Ovarian Tumor.*

Mrs. P. was the mother of five children, the youngest of whom was about seven years of age, in person rather spare, of a good constitution, intelligent, firm, and morally courageous.

The tumor was of three years' growth or more, having been accidentally discovered when when it was about the size of a turkey's egg, in crowding between a bed post and the wall of the room. For a year or two after, it gave her no alarm, or scarcely any anxiety; since it was neither painful nor tender, nor was its moderate increase in size calculated to excite serious apprehensions for the future; so that its existence was not made known to her physician until within eight or nine months of the day of the operation. During the last three or four months the tumor had increased so rapidly that she appeared like a woman far advanced in pregnancy. At last she was so much incommoded as to be entirely prevented from sleeping in the horizontal position.

After inquiring into the history of the case, and making a careful examination of the tumor, the general health and condition of the patient, as to the existence or non existence of any other disease, Dr. Munroe and myself agreed that it was a favorable case for the operation of extirpation. However, before proceeding in a matter of such vast importance to the patient and her friends, to say nothing of my own professional reputation, and that of the practice of the surgical art, I deemed it prudent to solicit a general consultation on Saturday, at which were present Dr. Munroe, Prof. E. Emmons, Prof. J. McNoughton, Prof. J. H. Armsby and Prof. Thos. Hun, my colleagues in the Albany Medical College, who came to the unanimous conclusion, after each had made a critical examination, that if an operation for an attempt at removal was ever warrantable in cases of this kind, this was a peculiarly favorable one.

The tumor was in the main smooth, though apparently lobulated, or having in one or two places a strictured or depressed line; and most of the bulk of it seemed to be made up of fluid, although there were one or two points apparently as large as the fist, which felt like a soft solid. In the abdominal wall there did not appear to be any tenderness or soreness to the sense of touch, nor had the patient ever experienced any pain in the swelling or abdomen, excepting an uneasiness for a few weeks, which was the result of pressure and over-distension of the abdominal walls.

The bowels were thoroughly evacuated on Sunday, by a calomel purgative, and on Monday, December 10th, 1840, at one o'clock, the operation was performed in the presence, and by the aid and assistance of the gentlemen who have already been named as council in the case, as well as several medical students.

An incision was commenced about four inches above the umbilicus and carried in the line of the linea alba to near the pubis. The abdominal wall at the upper part was extremely thin, and by two or three strokes of the knife the tumor was readily brought into view. The wound was so extensive, being about 12 inches, and the tumor so much exposed, that it was readily discovered by the eye and hands to lie almost loose in the abdomen. To facilitate its extraction, a puncture was made in the front and lower part of it, by which nearly, or all of the water from the cyst was evacuated. The collapsed sac was followed down to its attachment to the right angle of the uterus, by which it was discovered that the ovarium had been dilated into a *monolocular*, or single sac, attached to the uterus only by the broad ligament and Fallopian tube, which when twisted upon itself, or gathered together was not larger than the little finger; around which, in mass, a three threaded ligature was very tightly applied, not merely by the strength of a strong man, but by one of my own hands superadded. This was applied very near to the substance of the uterus; and next, all the substance included in the ligature, was severed about half an inch from the point of its application. In using the sponge to cleanse out a trifle of blood that had fallen into the abdomen, the ligature was detached, when a brisk, or rather an alarming hemorrhage ensued. The uterus was seized upon, drawn up, and the part containing the severed vessel, which was of the size of a crow's quill, or larger, was secured between the thumb and finger of my assistant, Dr. Armsby, until I could pass an armed needle with double ligature, the four ends of which I tied each way. The first did not prove effective in completely arresting the flow of blood. Another needle was passed in the same manner and secured as before, which answered the purpose. The patient must have lost nearly a pint of arterial blood, most of which fell into the cavity of the abdomen, and after having been properly removed, the wound in the abdominal wall was closed by eight interrupted sutures, and the ligatures brought out at the lower part of the wound. The abdomen was supported with long adhesive straps, a large sheet folded for a compress, and over the whole a towel or swathe applied after the manner of its use in obsetrical practice.

The shock of the operation, and the loss of blood being so severe for the patient, it was difficult to keep life in her for several hours. However, at the end of six hours, re-action began to take place. Although she was in the horizontal position during the operation, yet she became faint; which rendered it necessary for an assistant to make pressure with his hands on the bowels, to aid in keeping up the circulation. She also experienced a degree of nausea, which I was then rather disposed to attribute to the use of chloroform. Some four or five days after, there was a disposition to retch, but not sufficient to amount to much vomiting. This I attributed to the sympathetic irritation existing between the uterus and stomach. I believe this was all quieted by a little infusion of columbo.

In the management of the case, I constantly and regularly used morphine, for some ten or twelve days, and did not attempt to disturb the bowels with physic for eight or nine days. In the mean time, the bladder became distended with urine, and the bowels with air. The former was relieved by the use of the catheter, and the latter, by passing up the rectum, a large gum-elastic tube. By the adoption of these measures, the patient obtained great relief, and continued to improve from day to day in a very satisfactory manner.

I think the sutures were removed on the eighth day, when union by the first intention was complete, at every point, except at the lower angle of the wound occupied by the ligatures, with which the divided arteries were severed. There was no redness, nor scarcely any tenderness in any part of the abdomen, except at the lower part or in the region of the uterus. The ligatures had all come away, and the patient had so far recovered her health and strength, as to be able to make a journey of over a hundred miles in one day, on the thirty-fourth day after the operation.

The sac, when distended with its fluid, weighed 18 pounds.

In conclusion, I would say that I am apprehensive that a successful case or two, may encourage us to operate too often; when the favorable prospect, to say the least, might be but little encouraging. A monolocular hydatid, or fluid tumor, is regarded as the most favorable for removal.

There should be no adhesions between the cyst and the wall of the abdomen, omentum, or intestines. The foot stalk or pedicle of the tumor should be small. I should prefer not to have the patient tapped at all, except as an exploring operation just as extirpation was about to take place. I would recommend to the surgeon to place his finger beneath the broad ligament or pedicle of the tumor, and divide with the knife upon it such part as would include the blood vessels, and as they bleed secure them by a small ligature. After these are properly secured, I would then divide the fallopian tube and the remainder of the folds of the peritonium, which make up the broad ligament, as far from the angle of the uterus as practicable, and instead of fetching the ligatures out through the abdominal wall, I would suggest to have them threaded into a long *sail-maker's needle*, and conveyed by the aid of one finger in the vagina, carried high up, either in front or back of the uterus; and with the fingers of the other hand dipping down in the brim and cavity of the pelvis, through the pelvic partition transfixing it into the vagina, and out at the os externum.

By this procedure a less direct communication would be left with the peritoneal cavity; and if any serum or blood should remain in the lower part of the peritoneal cavity, it would in the course of a few days readily find an exit by the side of the ligatures, "per vaginam." In my case I do not think there was the least peritoneal inflammation, except that which was caused by and situated immediately around the ligatures, where they had their exit from the abdominal wall. Why I would apply the ligature to the insulated blood-vessels, and as far as possible from the uturus, would be to avoid irritation and inflammation of that organ, and the fold of the peritoneal membrane, which forms the broad ligament. By this means the fallopian tube needs not be in the least irritated by coming in contact with a foreign substance.—*Transactions of the N. Y. State Medical Society.*

